# Molecular Profiling Defines Distinct Prognostic Subgroups in Childhood AML: A Report From the French ELAM02 Study Group

**DOI:** 10.1097/HS9.0000000000000031

**Published:** 2018-02-21

**Authors:** Alice Marceau-Renaut, Nicolas Duployez, Benoît Ducourneau, Myriam Labopin, Arnaud Petit, Alexandra Rousseau, Sandrine Geffroy, Maxime Bucci, Wendy Cuccuini, Odile Fenneteau, Philippe Ruminy, Brigitte Nelken, Stéphane Ducassou, Virginie Gandemer, Thierry Leblanc, Gérard Michel, Yves Bertrand, André Baruchel, Guy Leverger, Claude Preudhomme, Hélène Lapillonne

**Affiliations:** 1 CHU Lille, Laboratory of Hematology, Lille, France; 2 INSERM, UMR-S 1172, Lille, France; 3 CH Valenciennes, Laboratory of Hematology, Valenciennes, France; 4 INSERM, U938, CDR Saint-Antoine, UPMC Paris 6, Paris, France; 5 AP-HP, Pediatric Hematology and Oncology Department, Trousseau Hospital, Paris, France; 6 AP-HP, Department of Clinical Pharmacology and Clinical Research Unit of East of Paris, Saint Antoine Hospital, Paris, France; 7 AP-HP, Department of Cytogenetics, Saint-Louis Hospital, Paris, France; 8 AP-HP, Laboratory of Hematology, Robert Debré University Hospital, Paris, France; 9 INSERM U918, Centre Henri Becquerel, Institute for Research and Innovation in Biomedicine, University of Rouen, Rouen, France; 10 CHU Lille, Department of Pediatric Hematology-Oncology, Lille, France; 11 Pediatric Oncology Hematology Unit/CEREVANCE/CIC 1401, INSERM CICP, University Hospital of Bordeaux, Pediatric Hospital, Bordeaux, France; 12 Department of Pediatric Hematology/Oncology, University Hospital of Rennes, Rennes 1 University, Rennes, France; 13 AP-HP, Department of Pediatric Hematology and Immunology, Robert Debré University Hospital, Paris, France; 14 CHU Marseille La Timone, Department of Pediatric Hematology, Marseille, France; 15 Claude Bernard University, Lyon, France; 16 Hospices Civils de Lyon, Institute of Hematology and Oncology Pediatrics, Lyon, France; 17 AP-HP, Laboratory of Hematology, Trousseau Hospital, Paris, France

## Abstract

Despite major treatment improvements over the past decades, pediatric acute myeloid leukemia (AML) is still a life-threatening malignancy with relapse rates up to 30% and survival rates below 75%. A better description of the pattern of molecular aberrations in childhood AML is needed to refine prognostication in such patients. We report here the comprehensive molecular landscape using both high-throughput sequencing focused on 36 genes and ligation-dependent RT-PCR in 385 children with de novo AML enrolled in the prospective ELAM02 trial and we evaluated their prognostic significance. Seventy-six percent of patients had at least 1 mutation among the genes we screened. The most common class of mutations involved genes that control kinase signaling (61%) followed by transcription factors (16%), tumor suppressors (14%), chromatin modifiers (9%), DNA methylation controllers (8%), cohesin genes (5%), and spliceosome (3%). Moreover, a recurrent transcript fusion was detected in about a half of pediatric patients. Overall, CBF rearrangements, *NPM1* and double *CEBPA* mutations represented 37% of the cohort and defined a favorable molecular subgroup (3 years OS: 92.1%) while *NUP98* fusions, *WT1*, *RUNX1*, and *PHF6* mutations (15% of the cohort) segregated into a poor molecular subgroup (3 years OS: 46.1%). *KMT2A*-rearrangements (21% of the cohort) were associated with an intermediate risk. Despite some overlaps, the spectrum of molecular aberrations and their prognostic significance differ between childhood and adult AML. These data have important implications to contribute in refining risk stratification of pediatric AML and show the need for further validations in independent pediatric cohorts.

## Abstract 2

Supplemental Digital Content is available in the text

## Introduction

Approximately 20% of childhood acute leukemia is of myeloid origin. Acute myeloid leukemia (AML) is defined as a clonal disorder caused by stepwise accumulation of successive genetic defects. In recent years, the use of genetic data to inform disease classification and clinical practice has been an active field of research. Improvements in identifying such molecular and cytogenetic aberrations have revealed the heterogeneity of this group of diseases. Recurrent mutations and gene fusions have been shown to affect a wide range of genes that have been classified into 8 functional categories: kinase signaling, transcription factors, tumor suppressors, DNA methylation, chromatin modifiers, cohesin, spliceosome, and the *NPM1* gene.^1^ Consequently, some genetic alterations with major prognostic significance - such as inv(16)(p13.1q22)/*CBFB–MYH11*, *t*(8;21)(q22;q22)/*RUNX1–RUNX1T1*, single *NPM1* mutations and *CEBPA* double mutations (*CEBPA*dm) - have been implemented into the World Health Organization (WHO) classification of AML.^2^ Nevertheless, most of investigations are based on the study of large cohorts of adult AML patients^3^,^4^ while genetic profiles are known to be quite different between adults and children with AML.^5^ Moreover, despite major treatment improvements over the past decades, pediatric AML is still associated with relapse rates up to 30% and survival rates below 75%.^6^ In this context, a better description of the pattern of molecular aberrations in childhood AML remains a great challenge to refine prognostication and improve outcome in such patients.

We report here the comprehensive molecular landscape of a large and well-annotated cohort of de novo pediatric AML enrolled in the prospective ELAM02 trial and propose a new prognostic molecular classifier in this particular group of patients.

## Methods

### Patients

The present study focuses on 385 patients of the 438 children treated in the ELAM02 trial (Treating Patients with Childhood Acute Myeloid Leukemia with Interleukin-2; ClinicalTrials.gov NCT00149162). Patient selection was based on the availability of genomic DNA at AML diagnosis. Children aged 0 to 18 years with newly diagnosed AML were enrolled between March 2005 and December 2011. Acute promyelocytic leukemia, therapy-related AML and Down syndromes were excluded from the ELAM02 trial. The study was approved by the Ethics Committee of Saint-Antoine Paris University Hospital (Assistance Publique-Hôpitaux de Paris) and by the Institutional Review Board of the French Regulatory Agency and was conducted in accordance with the Declaration of Helsinki.

### Cytogenetic analyses and extensive fusion transcripts detection

Cytogenetic analyses were locally performed on bone marrow samples using R- or G-banding. Results were centrally reviewed and described in accordance with the International System for Human Cytogenetic Nomenclature. Karyotypes were classified as follows: CBF-rearranged [i.e., inv(16)(p13q22)/*t*(16;16)(p13;q22) and *t*(8;21)(q22;q22)], *KMT2A*-rearranged, normal karyotype, adverse [i.e., monosomy 7, *t*(6;9)(p23;q34), inv(3)(q21q26)/*t*(3;3)(q21;q26) and complex karyotype] and other aberrations. A complex karyotype was defined by the presence of 3 or more unrelated chromosome abnormalities. Furthermore, all diagnosis samples were screened for more than 50 recurrent gene rearrangements and *KMT2A*-partial tandem duplication (*KMT2A*-PTD) using ligation-dependent RT-PCR amplification assay (LD-RT-PCR) as previously described by Ruminy et al.^7^

### Mutational analysis

Genomic DNA from bone marrow aspirates at diagnostic was studied by high-throughput sequencing (HTS) of 36 genes recurrently mutated in myeloid malignancies. The studied panel included genes encoding proteins involved in kinase signaling [*CBL* (exons 8–9), *FLT3* (exon 20), *JAK2* (exons 12, 14, 16), *KIT* (exons 8–13, 17), *KRAS* (exons 2–3), *MPL* (exon 10), *NRAS* (exons 2–3), *PTPN11* (exons 3, 13), *SETBP1* (exon 4)], transcription factors [*CEBPA* (exon 1), *ETV6* (exons 1–8), *GATA1* (exon 2), *GATA2* (exons 2-6), *RUNX1* (exons 1–6)], tumor suppressors [*PHF6* (exons 2–10), *PTEN* (exons 5–7), *TP53* (exons 2–11), *WT1* (exons 7, 9)], chromatin modifiers [*ASXL1* (exons 11–12), *BCOR* (exons 2–15), *BCORL1* (exons 1–12), *EZH2* (exons 2–20)], DNA methylation [*DNMT3A* (exons 2–23), *IDH1* (exon 4), *IDH2* (exon 4), *TET2* (exons 3–11)], cohesin complex [*NIPBL* (exons 2–47), *RAD21* (exons 2–14), *SMC1A* (exons 1–25), *SMC3* (exons 1–29), *STAG2* (exons 3–35)], RNA splicing [*SF3B1* (exons 13–18), *SRSF2* (exon 1), *U2AF1* (exons 2, 6), *ZRSR2* (exons 1–11)] and *NPM1* (exon 11). Two distinct HTS technologies were used to allow direct cross validation. Firstly, libraries were prepared using the Ampliseq System according to the manufacturer's instructions and run on Ion Proton (Thermofisher, Waltham, MA, USA). Raw data were analyzed with both Torrent Browser (Thermofisher) and SeqNext (JSI Medical System, Los Angeles, CA, USA). Secondly, libraries were also prepared using the Haloplex Target Enrichment System (Agilent Technologies, Santa Clara, CA, USA) and run on MiSeq (Illumina, San Diego, CA, USA). Raw data were processed by SureCall (Agilent Technologies) and SeqNext (JSI Medical System). A high depth of coverage (>1500×) was obtained for all genes with both HTS technologies, allowing detection of mutations with a variant allele frequency (VAF) until 1%. Frameshift and nonsense variants were always considered as relevant mutations. Single nucleotide variants were retained in the absence of description into public databases of human polymorphisms, and effects on protein function were predicted with 6 established prediction tools: SIFT (Sorting Intolerant From Tolerant), PolyPhen-1, PolyPhen-2, MAPP (Multivariate Analysis of Protein Polymorphism), PhD-SNP (Predictor of human Deleterious Single Nucleotide Polymorphism), and SNAP (Screening for Non-Acceptable Polymorphisms).^8^ The presence of the *FLT3*-internal tandem duplication (ITD) was performed for all patients by fragment analysis as previously described.^9^

### Statistical methods

Event-free survival (EFS) and overall survival (OS) were estimated by the Kaplan–Meier method and compared by cause-specific hazard Cox models. EFS was measured from the date of diagnosis to the date of the first event (induction failure, relapse, or death) or to the date of last follow-up. Patients who failed to achieve complete remission (CR) were considered as failures at day 60. OS was measured from the date of diagnosis to the date of death from any cause or last follow-up. Data were analyzed and compared without censor at transplant for patients who received allogeneic stem cell transplantation in first CR. Comparisons between patient subgroups were performed by the Mann–Whitney test for continuous variables and by Chi-square or Fisher exact test for categorical variables. Hazard ratios (HRs) are given with 95% confidence interval (CI). Multivariate analyses assessing the independent effect of the covariates were performed using Cox proportional hazard model. Variables associated with the outcome and a *P*-value < 0.10 in univariate analysis or known as validated factors were included in the multivariable models. Then a backward and forward stepwise selection was performed. All *P*-values were 2-sided and values <0.05 were considered statistically significant. All statistical tests were performed with the SPSS 22.0 (IBM Corp., Armonk, NY) and R3.2.3 software packages (R Development Core Team, Vienna, Austria).

## Results

### Patients’ characteristics at diagnosis

**Figure 1:**
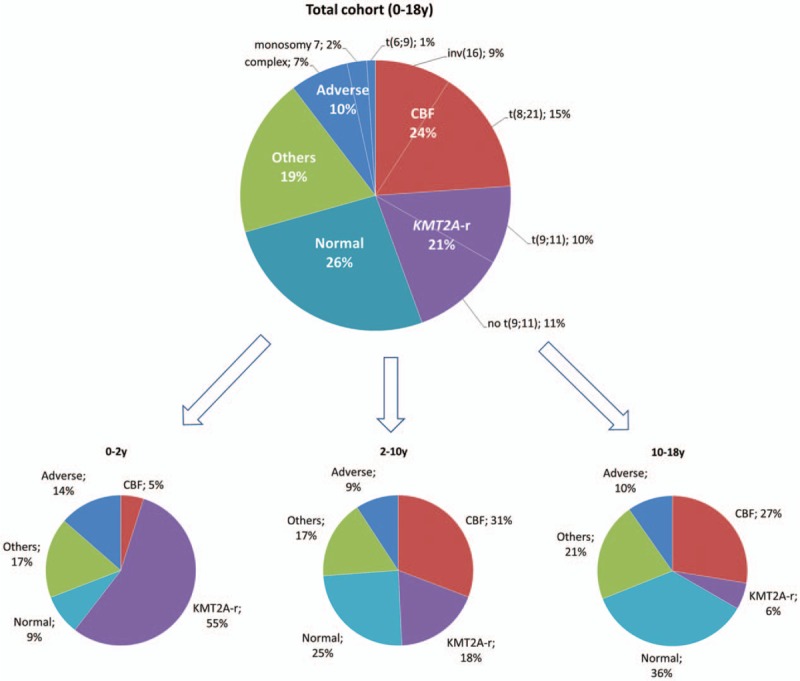
**Distribution of the cytogenetic subgroups in the studied cohort and according to age classes**.

Among the 385 patients in this study, 210 were male and 175 were female. The median age at AML diagnosis was 8.6 years (range, 0–18) and the median white blood cell (WBC) count was 16.6 × 10^9^/L (range, 0.40–575). The present cohort was not different from the entire ELAM02 cohort (Supplemental Table S1, Supplemental Digital Content). The distribution in the cytogenetic subgroups was as follows: normal karyotype (n = 101, 26.2%), CBF-rearranged (n = 92, 24% including *t*(8;21): n = 57 and inv(16)/*t*(16;16): n = 35), *KMT2A* (*MLL*)-rearranged (n = 79, 21%), adverse karyotype (n = 40, 10% including complex karyotype: n = 27, monosomy 7: n = 9 and *t*(6;9): n = 4), and other aberrations (named “others” hereafter) (n = 73, 19%) (Supplemental Table S1, Supplemental Digital Content, and Fig. 1). Inv(3) or *t*(3;3) was not identified in the present study. Cytogenetic appeared significantly different according to age with younger children harboring more *KMT2A*-rearrangements while other cytogenetic subgroups increased with age, especially for CBF-rearrangements and normal karyotypes (Fig. 1).

### Molecular profiling in childhood AML and association with cytogenetic aberrations

Molecular analyses with HTS and LD-RT-PCR allowed the identification of 579 mutations involving 35 different genes as well as 191 fusion transcripts (23 different fusion genes) among 385 pediatric patients.

**Figure 2:**
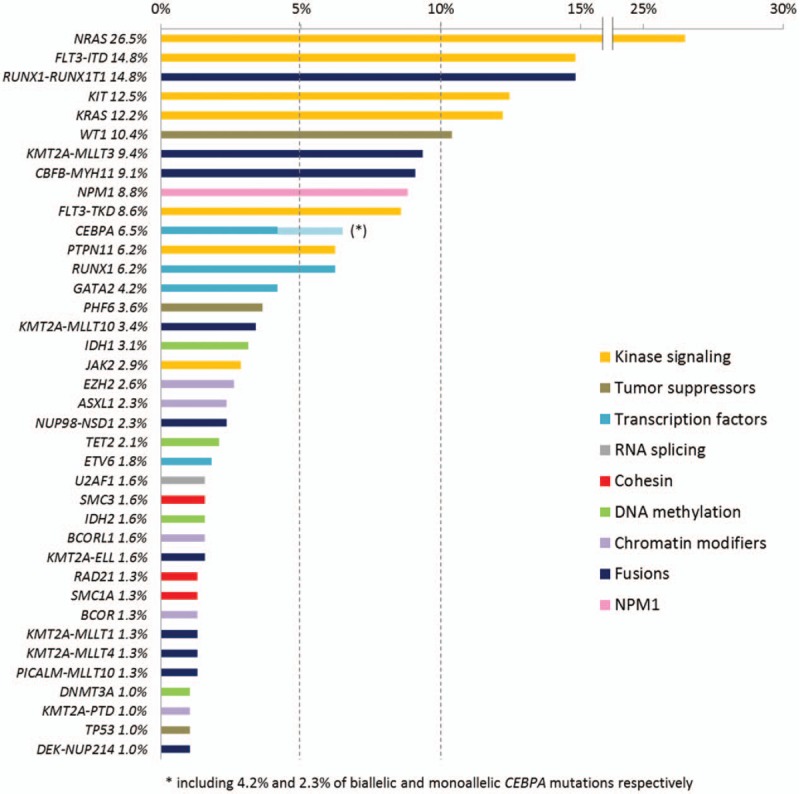
**Gene mutations and fusion transcripts frequencies in childhood AML.** Only aberrations detected with a frequency higher than 1% are shown.

Twenty-eight genes were mutated in more than 1% of our cohort but only 5 genes (*NRAS*, *FLT3*, *KIT*, *KRAS*, and *WT1*) were mutated in more than 10% (Fig. 2). The most common class of mutations involved genes that control kinase signaling (61% of the whole cohort) followed by transcription factors (16%), tumor suppressors (14%), chromatin modifiers (9%), DNA methylation controllers (8%), cohesin genes (5%), and spliceosome (3%). Overall, 76% of patients (292/385) had at least one mutation among the genes we examined. The mean number of mutated genes was 1.5 per patient (range, 0–5) with the highest rate of mutations in normal karyotype AML (mean 2.2; range, 0–5) and the lowest rate in *KMT2A*-rearranged AML (mean 0.7; range, 0–3). The mean number of mutated genes increased with age (means of 0.7, 1.5, and 1.9 for 0–2, 2–10, and 10–18 years, respectively, *P* < 0.001) mostly due to the different distribution of cytogenetic subgroups (Supplemental Figs. S1 and S2, Supplemental Digital Content).

The most frequent identified fusion transcripts were *RUNX1–RUNX1T1* (15%), *KMT2A–MLLT3* (9%), and *CBFB–MYH11* (9%). All other fusion transcripts were found in less than 5% of patients (Fig. 2 and Table S2, Supplemental Digital Content). *KMT2A* were found to be rearranged in 79 AML (21%) with 13 different partners among which *MLLT3* was by far the most common (n = 36, 46% of *KMT2A*-rearranged AML) followed by *MLLT10* (n = 13, 16%), *ELL* (n = 6, 8%), *MLLT1* (n = 5, 6%), and *MLLT4* (n = 5, 6%). Only 3 patients with *KMT2A*-rearrangement (identified by fluorescent in situ hybridization) had no identified partner. The cryptic *NUP98–NSD1* fusion was found in 9 patients (2.3% of the whole cohort) in which 5 had a normal karyotype.

Taken together, we identified at least 1 molecular aberration (mutations or fusion transcripts) in 344 (89%) out of 385 patients. Cytogenetics in the 41 remaining patients was distributed as follows: normal karyotype (n = 12), complex karyotype (n = 9), isolated monosomy 7 (n = 2), and other karyotype aberrations (n = 18).

**Figure 3:**
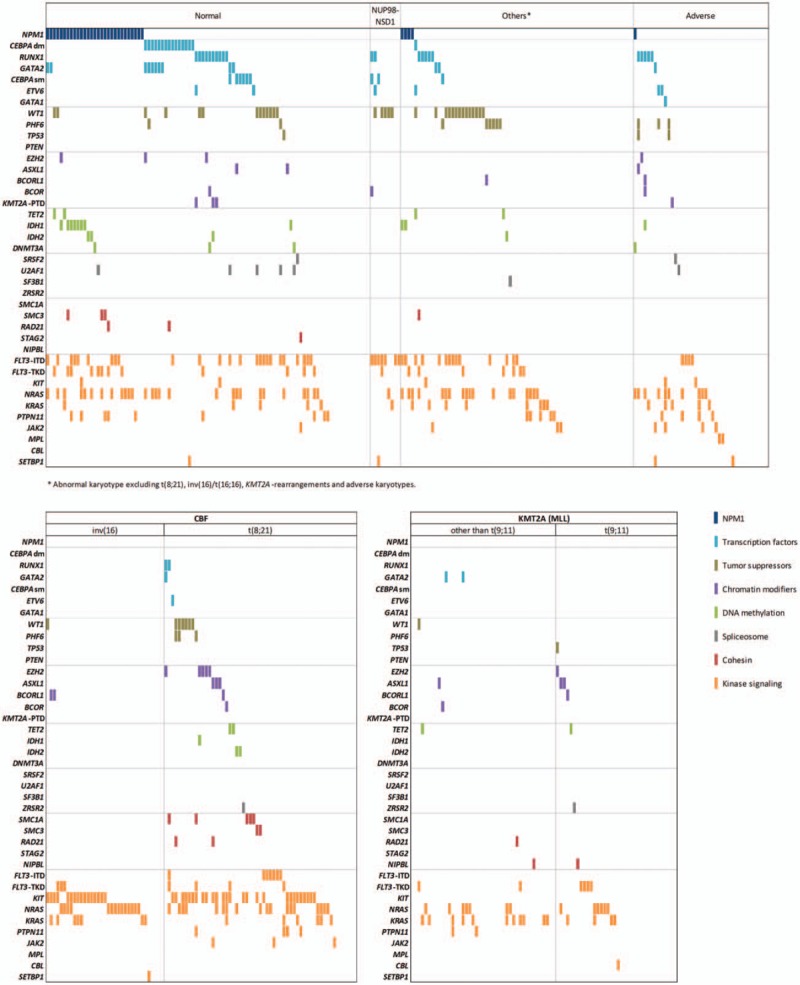
**Genomic landscape of childhood AML.** Each column represents the mutation pattern in one individual patient and each colored box represents a gene mutation. Genes are groups in 8 categories (in decreasing order): (1) *NPM1*; (2) transcription factors; (3) tumor suppressors; (4) chromatin modifiers; (5) DNA methylation; (6) spliceosome; (7) cohesin complex; (8) kinase signaling. The first row at the top represents the cytogenetic subgroup for each patient. Patients with *NUP98–NSD1* are distributed among normal karyotype (n = 5) and abnormal karyotype “other” (n = 4).

**Figure 4:**
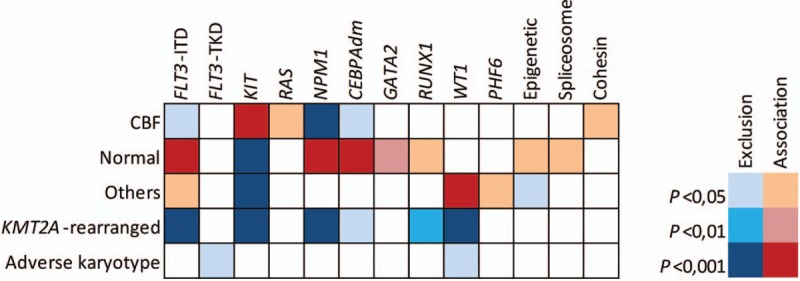
**Associations between mutations and cytogenetic subgroups.** Statistical significance was assessed using the Fisher exact test with adjustment with the Benjamini–Hochberg method.

**Figure 5:**
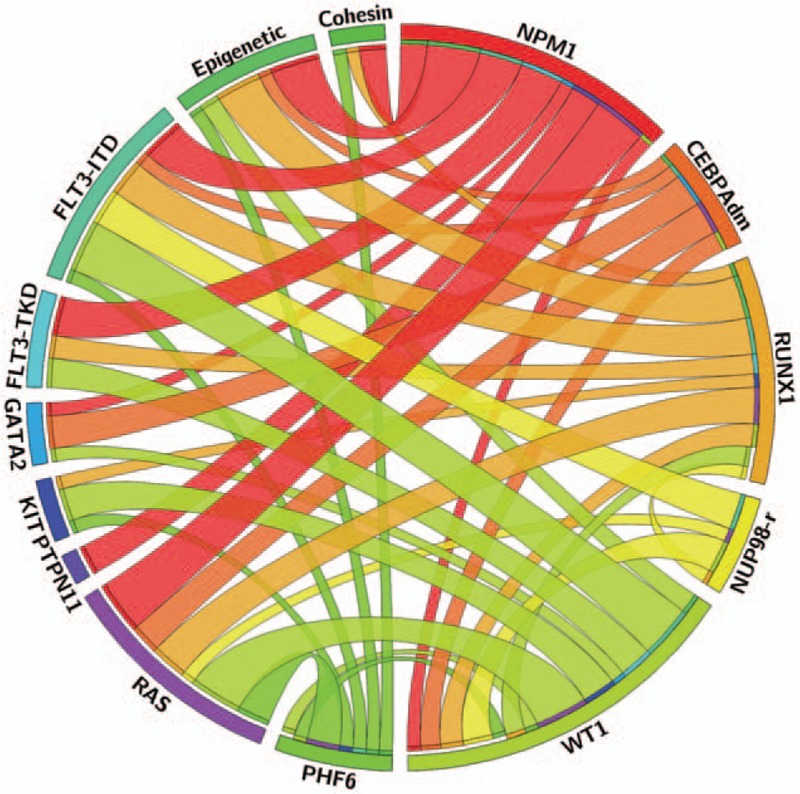
**Circos plot diagram illustrating the pairwise cooccurrence of molecular aberrations in childhood AML.** This figure was designed with the Circos online application (circos.ca).

Figure 3 depicts the interrelationship among the various mutations in cytogenetic subgroups. The mutational spectrum for the different age groups is provided in Supplemental Fig. S3 (Supplemental Digital Content). As expected, *NPM1* mutations, *FLT3*–ITD and *CEBPA* biallelic mutations (*CEBPA*dm) were associated with normal cytogenetics (*P* < 0.001 for each comparison) whereas *WT1* mutations were linked with the “other” subgroup (*P* < 0.001) (Fig. 4). CBF rearrangements were closely associated with *KIT* (*P* < 0.001), *RAS* (*P* = 0.012) and cohesin mutations (*P* = 0.024). Notably, mutations involving epigenetic regulators and cohesin genes were restricted to patients with *t*(8;21) AML while they were nearly absent in inv(16)/*t*(16;16) AML, as we described previously in a larger cohort of CBF AML including both pediatric and adult patients.^10^ On the other hand, no association was found in adverse cytogenetics and *KMT2A*-rearranged subgroups. We also investigated mutation cooccurrences showing that *NPM1* mutations were strongly associated with *FLT3*–ITD (*P* = 0.009), *FLT3*–TKD (*P* = 0.001) and mutations in epigenetic controllers (*P* < 0.001). *GATA2* mutations were significantly associated with *CEBPA*dm (*P* < 0.001), as previously described^11^,^12^ and *WT1* mutations appeared associated with *FLT3*–ITD (*P* < 0.001). *RUNX1* mutations were significantly associated with mutations in epigenetic controllers (*P* = 0.001) (Fig. 5). Considering that the *NUP98–NSD1* fusion has been associated with specific findings,^13^,^14^,^15^,^16^,^17^ the 9 positive patients were grouped together as a unique entity whatever karyotype aberrations for subsequent analyses. Consequently, a strong association was found between *NUP98–NSD1* fusion and *FLT3*–ITD (*P* < 0.001) and *WT1* mutations (*P* = 0.002).

### Impact of molecular abnormalities on complete remission rate and clinical outcome

Among the 385 patients included in this study, 350 (91%) achieved CR after 2 courses of intensive induction chemotherapy. In univariate analysis, *FLT3*–ITD, *WT1* mutations, WBC count higher than 30 × 10^9^/L, “other” cytogenetics and *NUP98* fusions were associated with more induction failures (Supplemental Table S3, Supplemental Digital Content). Despite the small number of cases, only the presence of a *NUP98* fusion remained associated with induction failure in multivariate analysis (*P* = 0.038) (Table 1). Characteristics of *NUP98*-rearranged cases are detailed below.

**Table 1:**
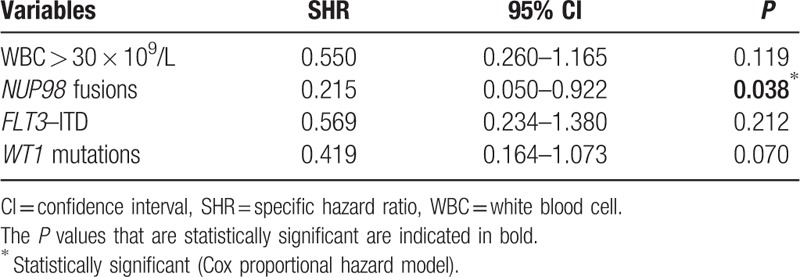
Multivariate Analysis for Complete Remission Achievement

At 3 years, EFS and OS for the whole cohort were estimated at 58.9% (95% CI: 54–63.9) and 76.1% (95% CI: 71.8–80.4) respectively with a median follow-up of 59 months. EFS and OS according to cytogenetic subgroups are presented in Supplemental Fig. S4 (Supplemental Digital Content). In univariate analysis, *NPM1* mutations, *CEBPA*dm and *KIT* mutations were associated with significant or a trend of higher OS and/or EFS (Supplemental Fig. S5A-F, Supplemental Digital Content). By contrast, *FLT3*–ITD, *WT1*, *RUNX1*, *PHF6*, and *NUP98*-rearrangements were associated with poorer OS and/or EFS (Supplemental Fig. S5G-P, Supplemental Digital Content).

Multivariate prognostic analyses for the HR are indicated in Table 2. Co-tested factors included *NPM1*, *CEBPA*dm, *FLT3*–ITD, *RUNX1*, *WT1*, and *PHF6* mutations as well as WBC count, cytogenetic subgroups, and NUP98 fusions. *KIT* mutations were excluded because of a strong association with CBF rearrangements. Five factors were demonstrated to be significantly associated with a higher risk of event by cause-specific hazard Cox models: WBC count higher than 30 × 10^9^/L (*P* = 0.005); *NUP98* fusions (*P* < 0.001); *FLT3*–*ITD* (*P* = 0.01): *WT1* mutations (*P* = 0.018) and adverse cytogenetics (*P* = 0.009). On the other hand, 4 factors were significantly associated with a lower risk of event: *NPM1* mutations (*P* = 0.009); *CEBPA*dm (*P* = 0.027); *CBF* rearrangements (*P* = 0.006); and *KMT2A* rearrangements (*P* = 0.021). A similar analysis for OS revealed 5 factors that have a negative impact: WBC count higher than 30 × 10^9^/L (*P* = 0.001); *WT1* mutations (*P* = 0.027); *RUNX1* mutations (*P* = 0.043); *PHF6* mutations (*P* = 0.038); and adverse cytogenetics (*P* < 0.001). On the other hand, 3 factors were shown to positively impact OS: *NPM1* mutations (*P* = 0.004), *CEBPA*dm (*P* = 0.042), and *CBF* rearrangements (*P* < 0.001).

**Table 2:**
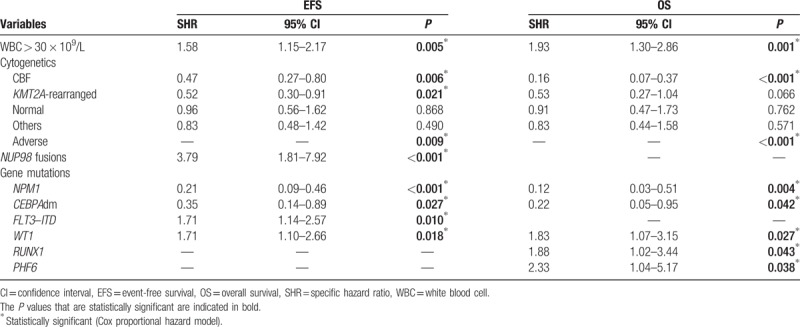
Multivariate Analysis for 3 Years EFS and OS

### *NUP98*-rearranged cases

*NUP98*-rearranged cases represented 2.6% of this cohort (10/385) with the fusion of *NUP98–NSD1* being found in 9 patients. The karyotype was normal in 5 patients and complex for the sole patient with *NUP98–JARID1A* fusion transcript. The 4 remaining patients belonged to the “other” cytogenetic subgroup. The median age was 9.9 years (range, 1.3–16.8) and median WBC count was 179.8 × 10^9^/L (range, 12.2–436). The most frequent mutations associated with this specific subgroup were *FLT3*–ITD (7/10), *WT1* (5/10), *CEBPA* (monoallelic mutation; 2/10), and *RUNX1* (2/10). Overall, *NUP98*-rearranged cases showed poor prognosis with a half of patients who did not achieve CR. At 3 years, EFS and OS in *NUP98*-rearranged cases were 10% (95% CI: 0–28.6) and 25% (95% CI: 0–54), respectively compared with 60.5% (95% CI: 55.5–65.5) and 77.3% (95% CI: 73.1–81.6) in *NUP98*-negative cases (Supplemental Fig. S5O-P, Supplemental Digital Content).

### Molecular classifier in childhood AML

**Figure 6:**
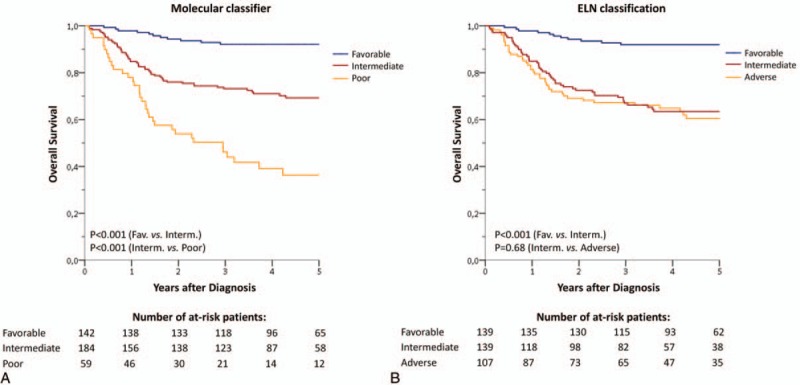
**Childhood AML outcome.** (A) Childhood AML outcome according to the molecular classifier. Favorable molecular risk: *RUNX1–RUNX1T1* or *CBFB–MYH11* or *NPM1* mutation or *CEBPA*dm; poor molecular risk: *NUP98* fusion or *RUNX1* or *WT1* or *PHF6* mutation; intermediate molecular risk (all others). (B) Childhood AML outcome according to the 2017 European LeukemiaNet (ELN) classification.^21^

Considering results from multivariate analysis and strong molecular markers validated among studies^6^,^18^,^19^,^20^ (i.e., *NPM1* mutations and *CEBPA*dm), we defined a molecular classifier, refining the prognosis in childhood AML. The molecular classifier was based on OS predictions and segregate AML into 3 groups (Supplemental Table S4, Supplemental Digital Content and Fig. 6A): favorable molecular risk (*RUNX1–RUNX1T1* or *CBFB–MYH11* or *NPM1* mutation or *CEBPA*dm, n = 142); poor molecular risk (*NUP98* fusion or *RUNX1* or *WT1* or *PHF6* mutation, n = 59); intermediate molecular risk (all others, n = 184). Patients who harbored both a CBF rearrangement and *WT1*, *RUNX1*, or *PHF6* mutations were included in the favorable subgroup. Neither karyotype nor other gene mutations were able to discriminate within patients in the intermediate molecular risk subgroup. At 3 years, OS was 92.1% (95% CI: 87.6–96.6, median not reached) for the favorable molecular risk subgroup, 73.2% (95% CI: 66.7–79.6, median not reached) for the intermediate molecular risk subgroup and 46.1% (95% CI: 33.1–59.2, median 2.33 years) for the poor molecular risk subgroup. Although *KMT2A*-rearrangements were associated with a trend of better outcome compared with non-*KMT2A*-rearranged cases from the intermediate subgroup, it did not reach statistical significance (*P* = 0.15). Consequently, *KMT2A*-rearrangements were not included in the classifier. The same results were observed when separating *KMT2A–MLLT3* rearrangements and other *KMT2A*-rearrangements. While *FLT3*–ITD was not retained as an independent prognostic factor for molecular classification, its cooccurrence in patients with poor molecular risk defined a subgroup of patients with the worst prognosis (3 years OS: 23.8% vs 58.8%; *P* = 0.024) (Supplemental Fig. S6, Supplemental Digital Content). By contrast, *FLT3*–ITD had no impact in the intermediate molecular risk group (P = 0.75) or in *NPM1*-mutated patients (*P* = 0.72).

Finally, the molecular classifier was compared to the 2017 European LeukemiaNet (ELN) classification^21^ which is currently used to stratify adult patients with AML. A total of 139 patients were classified in the favorable subgroup with both classifications. Only 3 *NPM1*-mutated-AML were classified as favorable according to the molecular classifier and as intermediate or adverse according to the ELN classification because of high *FLT3*–ITD ratio (n = 2) or complex karyotype (n = 1). Interestingly, the ELN classification fails to separate intermediate and adverse subgroups in our pediatric cohort (Fig. 6B, Supplemental Table S5, Supplemental Digital Content). Together, these data show that the ELN classification lacks of prognostic significance in childhood AML, especially in nonfavorable AML and the use of the present molecular classification could improve risk stratification in pediatric patients.

## Discussion

The better knowledge of molecular aberrations in AML has greatly improved the management of AML patients over the past decades. However, most of reported studies have focused on adult cohorts. The ELAM02 trial gave us the opportunity to investigate incidences and prognostic significances of molecular aberrations in childhood AML which currently remains a life-threatening malignancy with poor outcome compared to acute lymphoblastic leukemia.

The most common mutations involved genes controlling kinase signaling (especially *NRAS/KRAS*, *FLT3*–ITD, *KIT* mutations). These mutations concerned 61% of the whole cohort and were found in all cytogenetic subgroups. All but *FLT3*–ITD had no independent impact on outcome. To date, the prognostic significance of *FLT3*–ITD in pediatric AML remains controversial.^22^ In the present study, *FLT3*–ITD was associated with reduced EFS but did not influence OS in multivariate analysis in the whole cohort. Importantly, *FLT3*–ITD were found in heterogeneous diseases including *NPM1*-mutated or CBF AML which have shown to have a highly favorable outcome but also in *NUP98*-rearranged and *WT1*-mutated AML which are associated with poor prognosis. Among *NPM1*-mutated childhood AML, *FLT3*–ITD did not impact outcome in line with a previous study.^23^ Transcription factors were the second most common class of mutations (16% of the whole cohort). *CEBPAdm* and *RUNX1* mutations defined independent molecular subgroups of patients (4.2% and 6.2% respectively) associated with highly favorable and poor outcome respectively. Both mutations were mutual exclusive with *NPM1* mutations and occurred almost exclusively in normal karyotype-AML. By contrast, all other classes of mutations were found in less than 10% of patients. Importantly, while mutations within DNA-methylation-related genes (*DNMT3A*, *TET2*, *IDH1/2*) are highly prevalent in adult AML (together higher than 50%),^24^ only 8% of children with AML harbor such mutations, especially in normal karyotype-AML. Among normal karyotype-AML (n = 101/385), 3 patients harbored *DNMT3A* mutations (all at codon R882), 12 had *IDH1* mutations (codon R132), 4 had *IDH2* mutations (codon R140), and 3 had *TET2* mutations. These results are in line with a previous report from the Children's Oncology Group.^25^ Moreover, the systematic use of LD-RT-PCR allowed the detection of recurrent transcript fusions in about a half of pediatric patients. Fusions involving 1 of the 2 CBF subunits or the *KMT2A* gene were found in 24% and 21% of patients respectively. Among *KMT2A*-rearranged cases, the *KMT2A–MLLT3* fusion was by far the most common, representing nearly the half of *KMT2A* fusions. While CBF rearrangements were associated with a favorable prognosis, *KMT2A* rearrangements were associated with an intermediate outcome in the present study. *KMT2A–MLLT3* fusion did not show a better prognosis than other *KMT2A* rearrangements in line with a recent large retrospective study of *KMT2A*-rearranged pediatric AML.^26^

In accordance with previous reports,^6^,^18^,^19^,^20^,^27^ CBF rearrangements, *NPM1* mutations and *CEBPA*dm defined a particular subgroup with good prognosis. Together, these aberrations were found in more than one third of childhood AML. By contrast, *NUP98* fusions were associated with the worse prognosis, mostly due to induction failures. Other aberrations associated with poor outcome included *RUNX1*, *PHF6*, and *WT1* mutations. In a previous report by the Children's Oncology Group, *WT1* mutations were shown to be an independent factor of poor prognosis both on EFS and OS.^28^ Interestingly, AML with *RUNX1* mutations has been added to the last WHO classification as a provisional entity,^2^ considering they represent a biologically distinct group with a possibly worse prognosis in adults AML.^29^ Our results show that *RUNX1* mutations also defined a distinct subgroup with poor outcome in childhood AML. Finally, *PHF6* mutations are a rare event in childhood AML and to our knowledge, their prognosis impact has not been reported in a large series.^30^ Importantly, by contrast to the adult-based-ELN classification, the present molecular classification identified a group of pediatric patients with particular poor prognosis. Moreover, the cooccurrence of *FLT3*–ITD in this subgroup identified patients with the worst outcome. This result remains of great interest in the context of FLT3 inhibitors use.

In conclusion, we reported the comprehensive genomic landscape of a large cohort of pediatric de novo AML enrolled in the ELAM02 trial and proposed a prognostic classification based on gene mutations and fusions in this particular group of patients. Despite some overlaps between childhood and adult AML, pediatric patients harbored a different pattern of molecular aberrations, especially with fewer mutations within epigenetic-related genes. We confirmed the favorable-risk group including CBF fusions, *NPM1* mutations, and *CEBPA* biallelic mutations and refined the poor-risk group including *RUNX1*, *WT1*, and *PHF6* mutations as well as *NUP98* fusions. *KMT2A*-rearranged AML were included in the intermediate-risk group with no difference between *KMT2A–MLLT3* and other *KMT2A* fusions in this study. Overall, these results have important implications to contribute in refining risk stratification of pediatric AML and show the need for further validations in independent pediatric cohorts.

## Supplementary Material

Supplemental Digital Content

## References

[1] Döhner H Weisdorf DJ Bloomfield CD Acute myeloid leukemia. *N Engl J Med* 2015; 373:1136–1152. 26376137 10.1056/NEJMra1406184

[2] Arber DA Orazi A Hasserjian R The 2016 revision to the World Health Organization classification of myeloid neoplasms and acute leukemia. *Blood* 2016; 127:2391–2405. 27069254 10.1182/blood-2016-03-643544

[3] Patel JP Gönen M Figueroa ME Prognostic relevance of integrated genetic profiling in acute myeloid leukemia. *N Engl J Med* 2012; 366:1079–1089. 22417203 10.1056/NEJMoa1112304 PMC3545649

[4] Papaemmanuil E Gerstung M Bullinger L Genomic classification and prognosis in acute myeloid leukemia. *N Engl J Med* 2016; 374:2209–2221. 27276561 10.1056/NEJMoa1516192 PMC4979995

[5] Creutzig U Zimmermann M Reinhardt D Changes in cytogenetics and molecular genetics in acute myeloid leukemia from childhood to adult age groups: genetics in AML covering all age groups. *Cancer* 2016; 122:3821–3830. 27529519 10.1002/cncr.30220

[6] Zwaan CM Kolb EA Reinhardt D Collaborative efforts driving progress in pediatric acute myeloid leukemia. *J Clin Oncol* 2015; 33:2949–2962. 26304895 10.1200/JCO.2015.62.8289 PMC4567700

[7] Ruminy P Marchand V Buchbinder N Multiplexed targeted sequencing of recurrent fusion genes in acute leukaemia. *Leukemia* 2016; 30:757–760. 26139430 10.1038/leu.2015.177

[8] Bendl J Stourac J Salanda O PredictSNP: robust and accurate consensus classifier for prediction of disease-related mutations. *PLoS Comput Biol* 2014; 10:e1003440. 24453961 10.1371/journal.pcbi.1003440 PMC3894168

[9] Balsat M Renneville A Thomas X Postinduction minimal residual disease predicts outcome and benefit from allogeneic stem cell transplantation in acute myeloid leukemia with NPM1 mutation: a study by the Acute Leukemia French Association Group. *J Clin Oncol* 2016; 35:185–193. 28056203 10.1200/JCO.2016.67.1875

[10] Duployez N Marceau-Renaut A Boissel N Comprehensive mutational profiling of core binding factor acute myeloid leukemia. *Blood* 2016; 127:2451–2459. 26980726 10.1182/blood-2015-12-688705 PMC5457131

[11] Greif PA Dufour A Konstandin NP GATA2 zinc finger 1 mutations associated with biallelic CEBPA mutations define a unique genetic entity of acute myeloid leukemia. *Blood* 2012; 120:395–403. 22649106 10.1182/blood-2012-01-403220

[12] Marceau-Renaut A Guihard S Castaigne S Classification of CEBPA mutated acute myeloid leukemia by GATA2 mutations. *Am J Hematol* 2015; 90:E93–E94. 25611491 10.1002/ajh.23949

[13] Akiki S Dyer SA Grimwade D NUP98-NSD1 fusion in association with FLT3-ITD mutation identifies a prognostically relevant subgroup of pediatric acute myeloid leukemia patients suitable for monitoring by real time quantitative PCR. *Genes Chromosomes Cancer* 2013; 52:1053–1064. 23999921 10.1002/gcc.22100

[14] Fasan A Haferlach C Alpermann T A rare but specific subset of adult AML patients can be defined by the cytogenetically cryptic NUP98-NSD1 fusion gene. *Leukemia* 2013; 27:245–248. 22945772 10.1038/leu.2012.230

[15] Hollink IHIM van den Heuvel-Eibrink MM Arentsen-Peters STCJM NUP98/NSD1 characterizes a novel poor prognostic group in acute myeloid leukemia with a distinct HOX gene expression pattern. *Blood* 2011; 118:3645–3656. 21813447 10.1182/blood-2011-04-346643

[16] Ostronoff F Othus M Gerbing RB NUP98/NSD1 and FLT3/ITD coexpression is more prevalent in younger AML patients and leads to induction failure: a COG and SWOG report. *Blood* 2014; 124:2400–2407. 25145343 10.1182/blood-2014-04-570929 PMC4192751

[17] Shiba N Ichikawa H Taki T NUP98-NSD1 gene fusion and its related gene expression signature are strongly associated with a poor prognosis in pediatric acute myeloid leukemia. *Genes Chromosomes Cancer* 2013; 52:683–693. 23630019 10.1002/gcc.22064

[18] Creutzig U van den Heuvel-Eibrink MM Gibson B Diagnosis and management of acute myeloid leukemia in children and adolescents: recommendations from an international expert panel. *Blood* 2012; 120:3187–3205. 22879540 10.1182/blood-2012-03-362608

[19] Matsuo H Kajihara M Tomizawa D Prognostic implications of CEBPA mutations in pediatric acute myeloid leukemia: a report from the Japanese Pediatric Leukemia/Lymphoma Study Group. *Blood Cancer J* 2014; 4:e226. 25014773 10.1038/bcj.2014.47 PMC4219441

[20] Hollink I Zwaan CM Zimmermann M Favorable prognostic impact of NPM1 gene mutations in childhood acute myeloid leukemia, with emphasis on cytogenetically normal AML. *Leukemia* 2009; 23:262–270. 19020547 10.1038/leu.2008.313

[21] Döhner H Estey E Grimwade D Diagnosis and management of AML in adults: 2017 ELN recommendations from an international expert panel. *Blood* 2017; 129:424–447. 27895058 10.1182/blood-2016-08-733196 PMC5291965

[22] Wu X Feng X Zhao X Prognostic significance of FLT3-ITD in pediatric acute myeloid leukemia: a meta-analysis of cohort studies. *Mol Cell Biochem* 2016; 420:121–128. 27435859 10.1007/s11010-016-2775-1

[23] Hollink IHIM Zwaan CM Zimmermann M Favorable prognostic impact of NPM1 gene mutations in childhood acute myeloid leukemia, with emphasis on cytogenetically normal AML. *Leukemia* 2008; 23:262–270. 19020547 10.1038/leu.2008.313

[24] Renneville A Abdelali RB Chevret S Clinical impact of gene mutations and lesions detected by SNP-array karyotyping in acute myeloid leukemia patients in the context of gemtuzumab ozogamicin treatment: results of the ALFA-0701 trial. *Oncotarget* 2013; 5:916–932. 10.18632/oncotarget.1536 PMC4011594 24659740

[25] Ho PA Kutny MA Alonzo TA Leukemic mutations in the methylation-associated genes DNMT3A and IDH2 are rare events in pediatric AML: a report from the Children's Oncology Group. *Pediatr Blood Cancer* 2011; 57:204–209. 21504050 10.1002/pbc.23179 PMC3115394

[26] Balgobind BV Raimondi SC Harbott J Novel prognostic subgroups in childhood 11q23/MLL-rearranged acute myeloid leukemia: results of an international retrospective study. *Blood* 2009; 114:2489–2496. 19528532 10.1182/blood-2009-04-215152 PMC2927031

[27] Pollard JA Alonzo TA Gerbing RB Prevalence and prognostic significance of KIT mutations in pediatric patients with core binding factor AML enrolled on serial pediatric cooperative trials for de novo AML. *Blood* 2010; 115:2372–2379. 20056794 10.1182/blood-2009-09-241075 PMC2845895

[28] Ho PA Zeng R Alonzo TA Prevalence and prognostic implications of WT1 mutations in pediatric acute myeloid leukemia (AML): a report from the Children's Oncology Group. *Blood* 2010; 116:702–710. 20413658 10.1182/blood-2010-02-268953 PMC2918327

[29] Gaidzik VI Bullinger L Schlenk RF RUNX1 mutations in acute myeloid leukemia: results from a comprehensive genetic and clinical analysis from the AML Study Group. *J Clin Oncol* 2011; 29:1364–1372. 21343560 10.1200/JCO.2010.30.7926

[30] Rooij JD Heuvel-Eibrink MM Rijdt NK PHF6 mutations in paediatric acute myeloid leukaemia. *Br J Haematol* 2016; 175:967–971. 27885656 10.1111/bjh.13891

