# Supplementary material for: Molecular Profiling Defines Distinct Prognostic Subgroups in Childhood AML: A Report From the French ELAM02 Study Group
Source: Hemasphere. 2018 Feb 21;2(1):e31. doi: 10.1097/HS9.0000000000000031 (PMC6745946; doi:10.1097/HS9.0000000000000031)

**Appendix**

**Molecular profiling defines distinct prognostic subgroups in childhood acute myeloid leukemia: a report from the French ELAM02 study group**

Alice Marceau-Renaut^*^, Nicolas Duployez^*^, Benoît Ducourneau, Myriam Labopin, Arnaud Petit, Alexandra Rousseau, Sandrine Geffroy, Maxime Bucci, Wendy Cuccuini, Odile Fenneteau, Philippe Ruminy, Brigitte Nelken, Stéphane Ducassou, Virginie Gandemer, Thierry Leblanc, Gérard Michel, Yves Bertrand, André Baruchel, Guy Leverger, Claude Preudhomme and Hélène Lapillonne

[**Appendix Table S1:** Patients' characteristics at AML diagnosis. 3](#_Toc499821717)

[**Appendix Table S2:** Fusion transcripts identified by LD-RTPCR. 3](#_Toc499821718)

[**Appendix Table S3:** Patients’ characteristics according to complete remission status after two courses of intensive induction chemotherapy. 4](#_Toc499821719)

[**Appendix Table S4:** 3-years OS for molecular classifier subgroups. 5](#_Toc499821720)

[**Appendix Table S5:** 3-years OS according to the 2017 ELN risk stratification. 5](#_Toc499821721)

[**Appendix Figure S1:** Number of mutations according to age classes. 6](#_Toc499821722)

[**Appendix Figure S2:** Number of mutations according to cytogenetics. 6](#_Toc499821723)

[**Appendix Figure S3:** Genomic landscape of childhood AML according to age groups.. 7](#_Toc499821724)

[**Appendix Figure S4:** Childhood AML outcome according to cytogenetic subgroups. 8](#_Toc499821725)

[**Appendix Figure S5:** Childhood AML outcome according to gene mutations. 9](#_Toc499821726)

[**Appendix Figure S6:** Impact of *FLT3-ITD* status in childhood AML with poor molecular risk. 11](#_Toc499821727)

# Appendix Table S1: Patients' characteristics at AML diagnosis (total ELAM02 cohort vs. studied cohort).

| **Characteristics** | | | **No. (%)**  **ELAM02 study (n=438)** | **No. (%)**  **Present study (n=385)** |
| --- | --- | --- | --- | --- |
| Sex | |  |  |  |
|  | Male | | 238 (54.3) | 210 (54.5) |
|  | Female | | 200 (45.7) | 175 (45.5) |
| Median age, years (range) | | | 8.2 (0-18) | 8.6 (0-18) |
| Median WBC count, x10^9^/L (range) | | | 15.4 (0.40-575) | 16.6 (0.40-575) |
| Cytogenetics | | |  |  |
|  | CBF-rearranged | | 97 (22.1) | 92 (23.9) |
|  |  | inv(16)/t(16;16) | 36 (8.2) | 35 (9.1) |
|  |  | t(8;21) | 61 (13.9) | 57 (14.8) |
|  | *KMT2A* (*MLL*)-rearranged | | 95 (21.7) | 79 (20.5) |
|  |  | t(9;11) | 40 (9.1) | 36 (9.4) |
|  |  | no t(9;11) | 55 (12.6) | 43 (11.1) |
|  | Normal karyotype | | 109 (24.9) | 101 (26.2) |
|  | Others | | 77 (17.6) | 73 (19) |
|  | Adverse | | 55 (12.5) | 40 (10.4) |
|  |  | Complex karyotype | 39 (8.9) | 27 (7) |
|  |  | monosomy 7 | 11 (2.5) | 9 (2.3) |
|  |  | t(6;9) | 5 (1.1) | 4 (1) |
| Remission | | | 398 (90.9) | 350 (90.9) |
| EFS, 3 years | | | 56% (49.7-63.6) | 58.9% (54-63.9) |
| OS, 3 years | | | 71.5% (65-78) | 76.1% (71.8-80.4) |

# Appendix Table S2: Fusion transcripts identified by LD-RTPCR.

| **Fusion transcripts** | **Corresponding cytogenetic aberration** | **Number of positive patients, n (%)** |
| --- | --- | --- |
| *RUNX1-RUNX1T1* | t(8;21)(q22;q22) | 57 (14.8%) |
| *KMT2A-MLLT3* | t(9;11)(p22;q23) | 36 (9.4%) |
| *CBFB-MYH11* | inv(16)(p13q22) or t(16;16)(p13;q22) | 35 (9.1%) |
| *KMT2A-MLLT10* | t(10;11)(p12;q23) | 13 (3.4%) |
| *NUP98-NSD1* | t(5;11)(q35;p15.5) | 9 (2.3%) |
| *KMT2A-ELL* | t(11;19)(q23;p13.1) | 6 (1.6%) |
| *KMT2A-MLLT1* | t(11;19)(q23;p13.3) | 5 (1.3%) |
| *KMT2A-MLLT4* | t(6;11)(q27;q23) | 5 (1.3%) |
| *PICALM-MLLT10* | t(10;11)(p13;q21) | 5 (1.3%) |
| *DEK-NUP214* | t(6;9)(p22;q34) | 4 (1.0%) |
| *KMT2A-MLLT11* | t(1;11)(q21;q23) | 3 (0.8%) |
| *KMT2A-SEPT6* | t(X;11)(q24;q23)) | 2 (0.5%) |
| *KAT6A-CREBBP* | t(8;16)(p11;p13) | 1 (0.3%) |
| *KMT2A-ABI1* | t(10;11)(p12;q23) | 1 (0.3%) |
| *KMT2A-ADARB2* | t(10;11)(p15.3;q23) | 1 (0.3%) |
| *KMT2A-EPS15* | t(1;11)(p32;q23) | 1 (0.3%) |
| *KMT2A-KIAA1524* | t(3;11)(q13.3;q23) | 1 (0.3%) |
| *KMT2A-PICALM* | t(11;11)(q14;q23) | 1 (0.3%) |
| *KMT2A-SEPT5* | t(11;22)(q23;q11.2) | 1 (0.3%) |
| *MN1-ETV6* | t(12;22)(p13;q12) | 1 (0.3%) |
| *MYB-GATA1* | t(X;6)(p11;q23) | 1 (0.3%) |
| *NUP98-JARID1A* | t(11;12)(p15;p13) | 1 (0.3%) |
| *RBM15-MKL1* | t(1;22)(p13;q13) | 1 (0.3%) |

# Appendix Table S3: Patients’ characteristics according to complete remission status after two courses of intensive induction chemotherapy.

| **Characteristics** | |  | **Total No.** | **CR** | **no CR** | **p-value** |
| --- | --- | --- | --- | --- | --- | --- |
| Sex (M/F) | |  | 210/175 | 187/163 | 23/12 | 0.164 |
| WBC>30**×**10^9^/L | |  | 148 | 128 | 20 | 0.017* |
| **Cytogenetics** | |  |  |  |  | 0.028* |
|  | CBF |  | 92 | 88 | 4 | 0.094 |
|  | *KMT2A*-rearranged | | 79 | 76 | 3 | 0.079 |
|  | Normal |  | 101 | 90 | 11 | 0.545 |
|  | Others | | 73 | 61 | 12 | 0.023* |
|  | Adverse |  | 40 | 35 | 5 | 0.389 |
| *NUP98* fusions | | | 10 | 5 | 5 | 0.001* |
| **Gene mutations** | | |  |  |  |  |
| Tyrosine kinase pathway | | | 236 | 213 | 23 | 0.792 |
|  | *FLT3-ITD* |  | 57 | 46 | 11 | 0.004* |
|  | *FLT3-TKD* |  | 33 | 31 | 2 | 0.754 |
|  | *KIT* |  | 48 | 46 | 2 | 0.286 |
|  | *NRAS* |  | 102 | 94 | 8 | 0.609 |
|  | *KRAS* |  | 47 | 42 | 5 | 0.598 |
|  | *CBL* |  | 1 | 1 | 0 | 1.000 |
|  | *PTPN11* |  | 24 | 22 | 2 | 1.000 |
|  | *JAK2* |  | 11 | 10 | 1 | 1.000 |
|  | *MPL* |  | 2 | 2 | 0 | 1.000 |
|  | *SETBP1* |  | 4 | 3 | 1 | 0.318 |
| Transcription Factors | | | 61 | 55 | 6 | 0.825 |
|  | *CEBPA* |  | 25 | 24 | 1 | 0.715 |
|  | *RUNX1* |  | 24 | 20 | 4 | 0.257 |
|  | *GATA2* |  | 16 | 16 | 0 | 0.380 |
|  | *GATA1* |  | 1 | 1 | 0 | 1.000 |
|  | *ETV6* |  | 7 | 6 | 1 | 0.490 |
| Epigenetic | |  | 58 | 54 | 4 | 0.629 |
| -Chromatin Modifiers | | | 33 | 33 | 0 | 0.058 |
|  | *ASXL1* |  | 9 | 9 | 0 | 1.000 |
|  | *EZH2* |  | 10 | 10 | 0 | 0.609 |
|  | *BCOR* |  | 5 | 5 | 0 | 1.000 |
|  | *BCORL1* |  | 6 | 6 | 0 | 1.000 |
| -DNA Methylation | | | 30 | 26 | 4 | 0.336 |
|  | *IDH1* |  | 12 | 11 | 1 | 1.000 |
|  | *IDH2* |  | 6 | 4 | 2 | 0.095 |
|  | *DNMT3A* |  | 4 | 4 | 0 | 1.000 |
|  | *TET2* |  | 8 | 7 | 1 | 0.537 |
| Tumor Suppressors | | | 54 | 44 | 10 | 0.018 |
|  | *WT1* |  | 40 | 31 | 9 | 0.005* |
|  | *TP53* |  | 4 | 4 | 0 | 1.000 |
|  | *PHF6* |  | 14 | 13 | 1 | 1.000 |
| Cohesin | |  | 19 | 19 | 0 | 0.239 |
|  | *SMC1A* |  | 5 | 5 | 0 | 1.000 |
|  | *SMC3* |  | 6 | 6 | 0 | 1.000 |
|  | *RAD21* |  | 5 | 5 | 0 | 1.000 |
|  | *STAG2* |  | 1 | 1 | 0 | 1.000 |
|  | *NIPBL* |  | 2 | 2 | 0 | 1.000 |
| Spliceosome | |  | 11 | 9 | 2 | 0.263 |
|  | *SRSF2* |  | 2 | 1 | 1 | 0.174 |
|  | *U2AF1* |  | 6 | 5 | 1 | 0.438 |
|  | *SF3B1* |  | 1 | 1 | 0 | 1.000 |
|  | *ZRSR2* |  | 2 | 2 | 0 | 1.000 |
| *NPM1* | |  | 34 | 34 | 0 | 0.058 |

# Appendix Table S4: 3-years OS for molecular classifier subgroups.

| Molecular classifier subgroups | | Number of patients | 3-years OS |
| --- | --- | --- | --- |
| Favorable | | **142** | **92.1% [95%CI: 87.6-96.6]** |
|  | *NPM1* mutations | 34 | 97.1% |
|  | *CEBPA* double mutations | 16 | 87.1% |
|  | CBF translocations | 92 | 91.2% |
| Intermediate | | **184** | **73.2% [95%CI: 66.7-79.6]** |
|  | *KMT2A*-rearranged | 79 | 79.7% |
|  | Other profiles | 105 | 68.2% |
| Poor | | **59** | **46.1% [95%CI: 33.1-59.2]** |
|  | *NUP98* fusions | 10 | 25.0% |
|  | *PHF6* mutations | 9 | 44.4% |
|  | *RUNX1* mutations | 20 | 59.6% |
|  | *WT1* mutations | 20 | 42.8% |
| Total | | **385** | **76.1% [95%CI: 71.8-80.4]** |

# Appendix Table S5: 3-years OS according to the 2017 ELN risk stratification.

| ELN classification | | Number of patients | 3-years OS |
| --- | --- | --- | --- |
|  | Favorable | 139 | 91.9% [95%CI: 87.4-96.5] |
|  | Intermediate | 139 | 67% [95%CI: 59.1-75] |
|  | Adverse | 107 | 67.2% [95%CI: 58.3-76.1] |

# Appendix Figure S1: Number of mutations according to age classes.


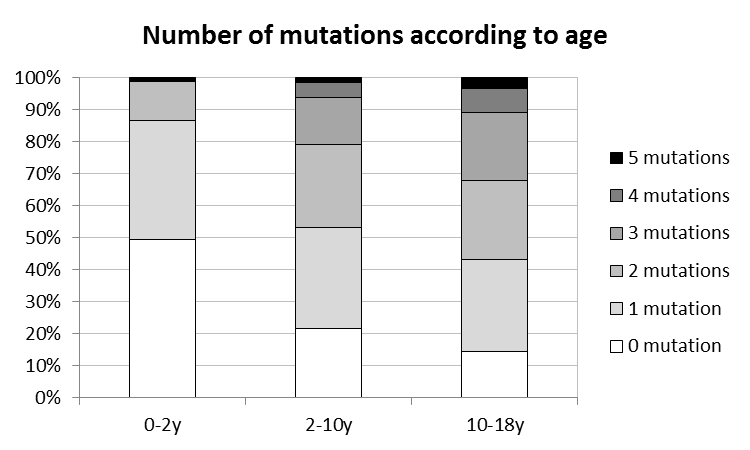


# Appendix Figure S2: Number of mutations according to cytogenetics.


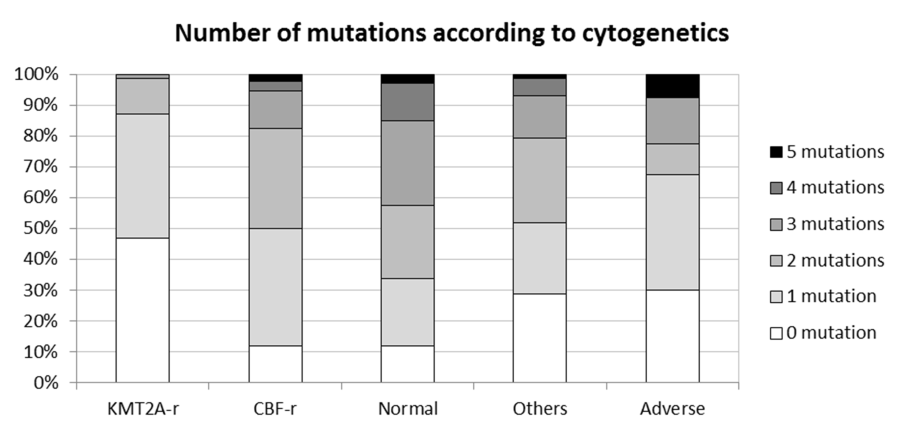


# Appendix Figure S3: Genomic landscape of childhood AML according to age groups (0-2 years; 2-10 years; 10 years or more). Each column represents the mutation pattern in one individual patient and each colored box represents a gene mutation.


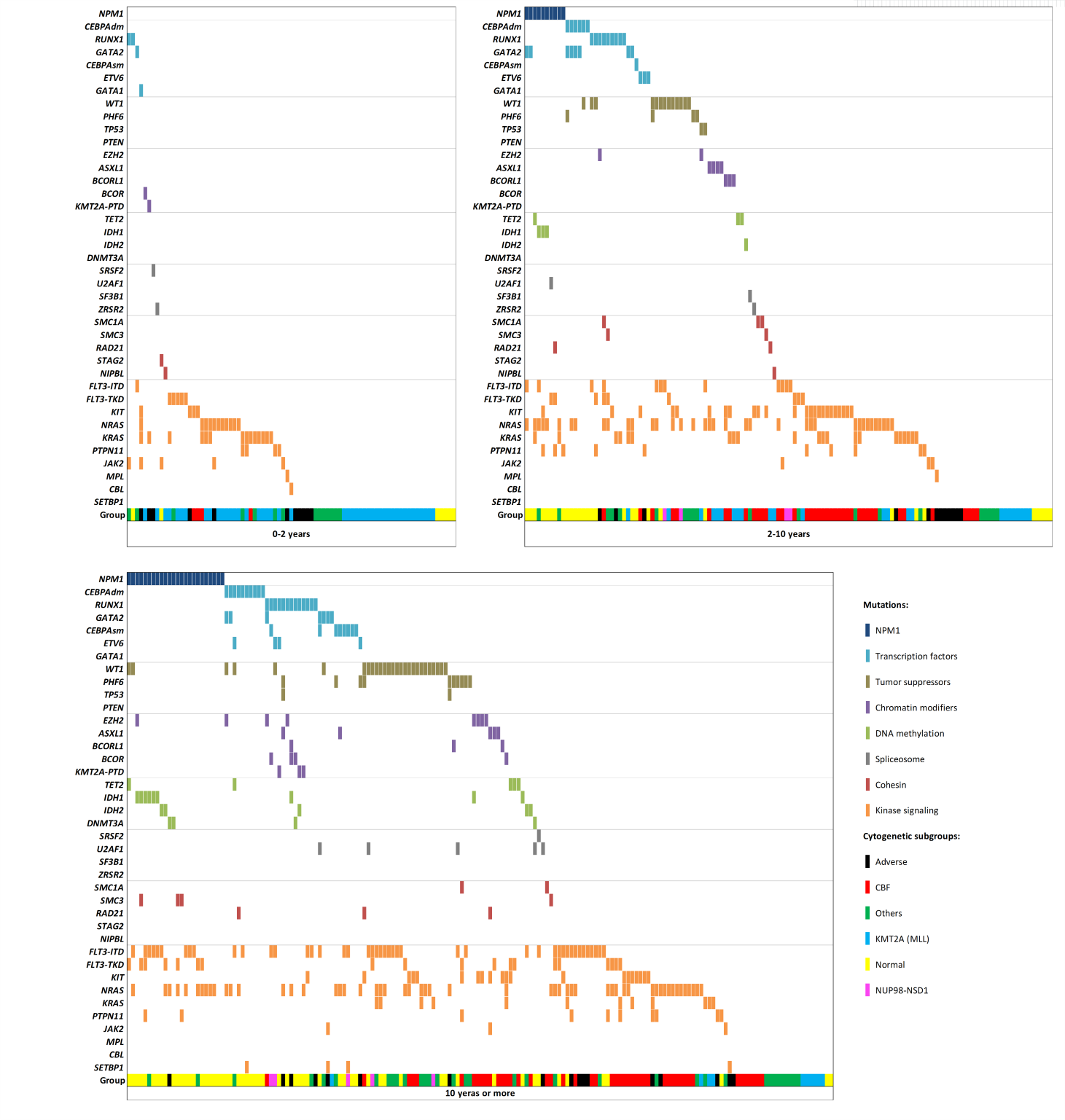


# Appendix Figure S4: Childhood AML outcome according to cytogenetic subgroups: normal karyotype (n=101, 26.2%), CBF-rearranged (n=92, 24%), *KMT2A*-rearranged (n=79, 21%), adverse karyotype (n=40, 10%) and other aberrations (n=73, 19%).


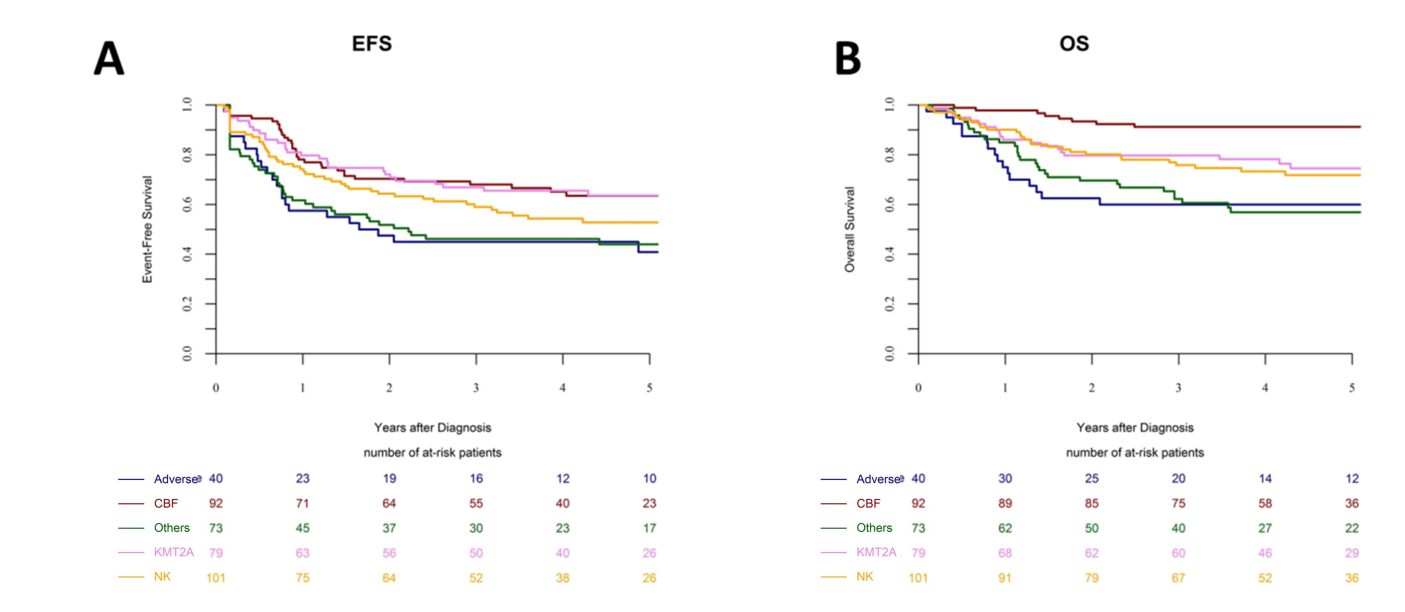


# Appendix Figure S5: Childhood AML outcome according to gene mutations. EFS and OS curves are shown for gene mutations which impact clinical outcome: *NPM1* (A-B), *CEBPAdm* (C-D), *KIT* (E-F), *FLT3-ITD* (G-H), *WT1* (I-J), *RUNX1* (K-L), *PHF6* (M-N) and *NUP98* fusions (O-P).


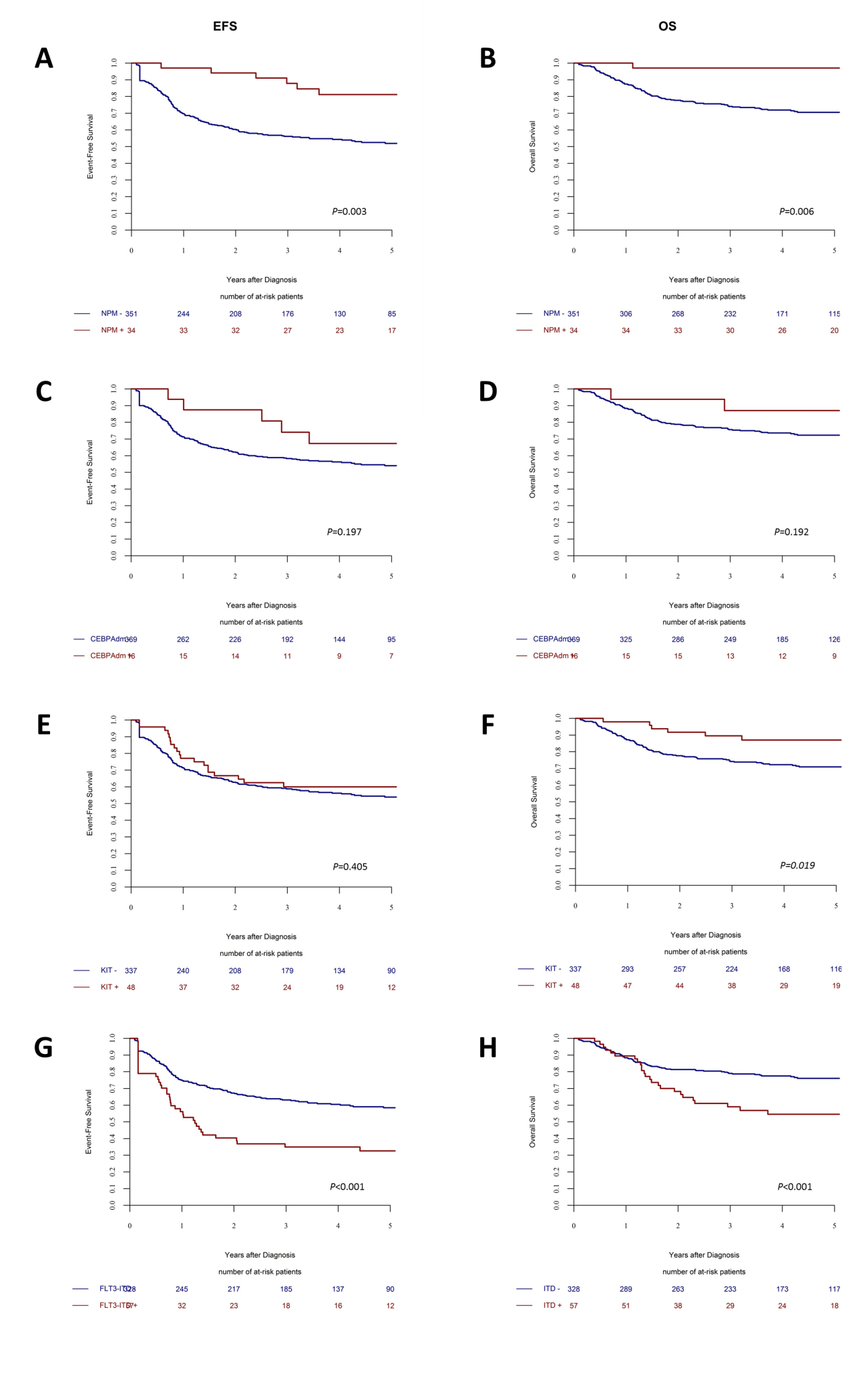


**(continued)**


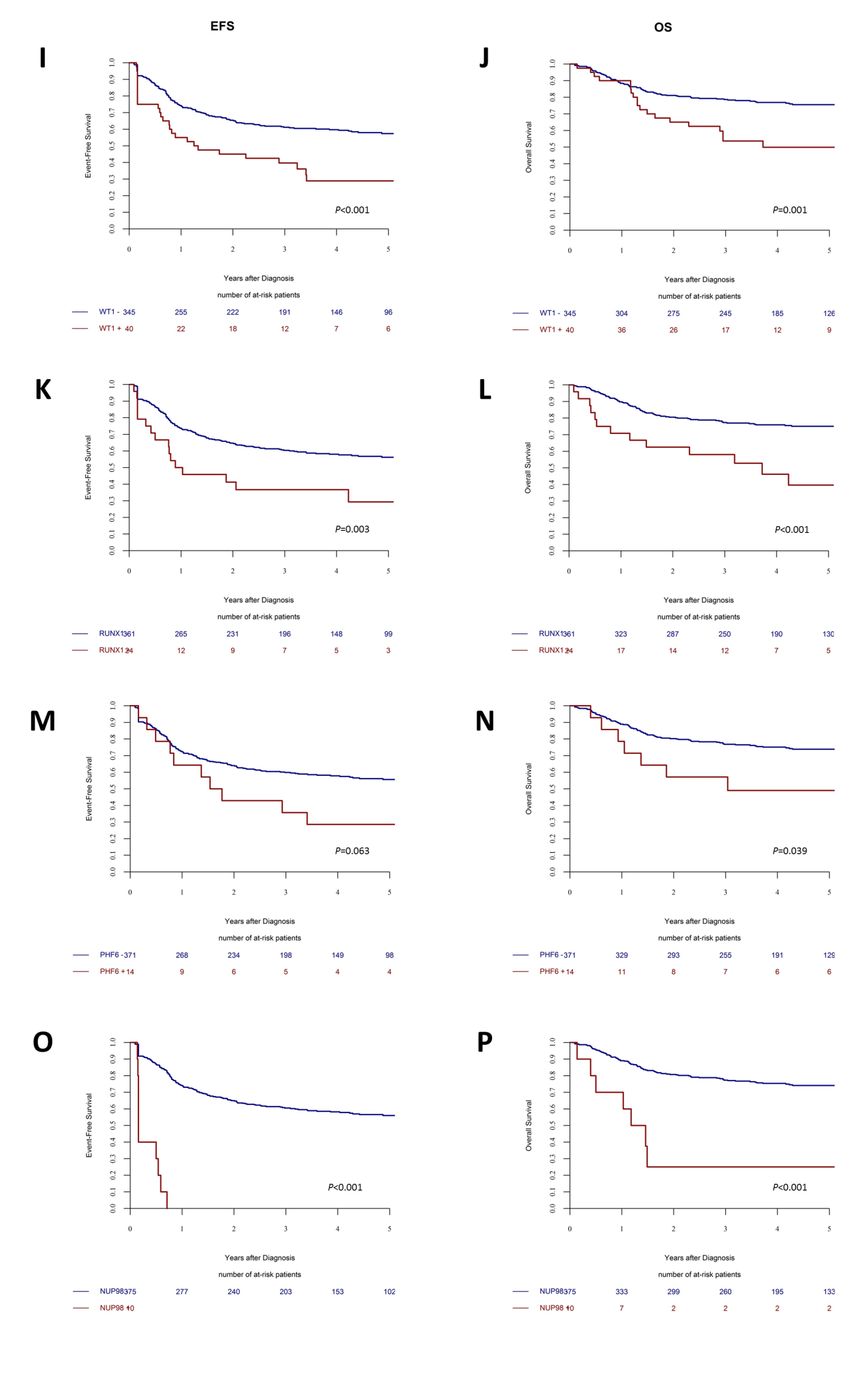


# Appendix Figure S6: Impact of *FLT3-ITD* status in childhood AML with poor molecular risk (*NUP98*-rearrangements, *WT1*, *RUNX1* or *PHF6* mutations). Among patients with poor molecular risk (n=59), 22 were positive for *FLT3*-ITD.


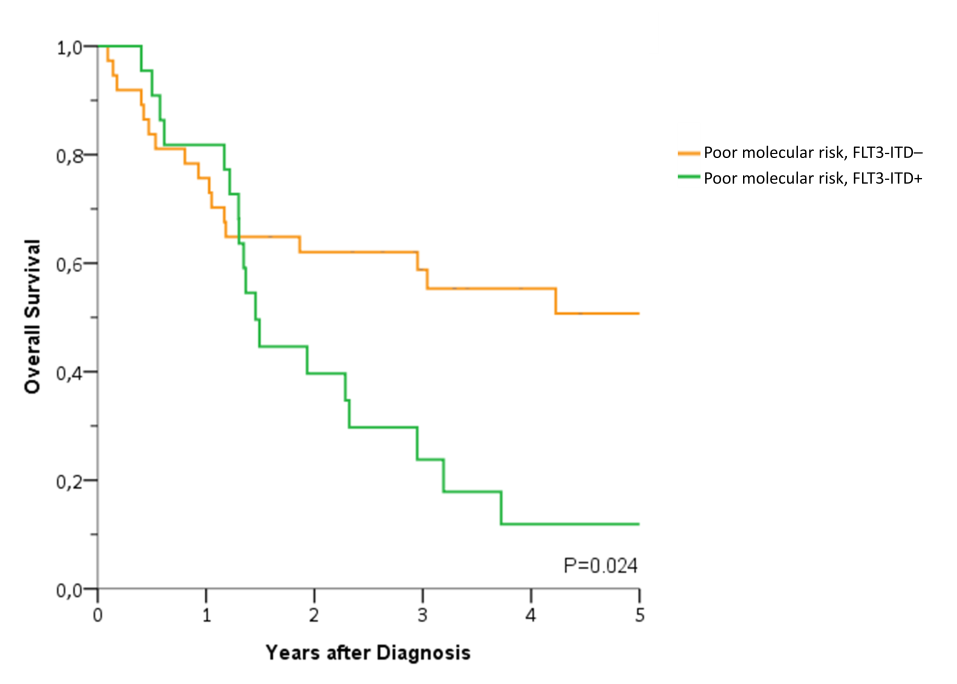

Supplement: Supplemental Digital Content [file hs9-2-e31-s001.docx]
